# Is the use of dual antiplatelet therapy following urgent and emergency coronary artery bypass surgery associated with increased risk of cardiac tamponade?

**Published:** 2021-03-13

**Authors:** Azar Hussain, Vassili Crispi, Shereen Ajab, Emmanuel Isaac, Ghazi Elshafie, Mahmoud Loubani

**Affiliations:** ^1^Department of Cardiothoracic Surgery, Castle Hill Hospital, Hull University Teaching Hospitals NHS Trust, Hull, UK; ^2^Hull York Medical School, Allam Medical Building, University of Hull, Hull, UK

**Keywords:** aspirin, clopidogrel, ticagrelor, pericardial effusion

## Abstract

**Background and aim:**

Cardiac tamponade is a recognized post-cardiac surgery complication, resulting in increased morbidity and mortality. The 2016 American College of Cardiology and American Heart Association Guidelines recommended the use of Dual Antiplatelet Therapy (DAPT) in the management of patients undergoing urgent or emergency coronary artery bypass grafting (CABG). The effect of DAPT on cardiac tamponade rates was investigated in comparison to aspirin monotherapy (AMT).

**Materials and methods:**

Prospectively collected data from a tertiary cardiac surgery center was analyzed to identify the patients who underwent urgent and emergency CABG between January 2015 and January 2018. The patients were categorized as aspirin monotherapy (AMT) and Dual Antiplatelet Therapy (DAPT) groups. The primary outcome was total cardiac tamponade rate and secondary outcomes were length of hospitalization and 30-days and 1-year mortality.

**Results:**

A total of 246 eligible patients were included across both arms and compared for confounding variables. Cardiac tamponade was observed in 9 (7.3%) and 8 (6.5%) of AMT and DAPT groups, respectively (P=0.802). The average hospital stay in days was similar in both groups (AMT=8.4 vs. DAPT=8.1, P=0.82), whereas tamponade patients experienced a significantly longer hospitalization when compared to non-tamponade patients (9.8 vs. 8.1 days, P=0.047). The 30-days and 1-year mortality were similar in both groups and were 0.8% and 1.6%, respectively.

**Conclusion:**

Overall, this study demonstrated that DAPT in urgent or emergency CABG patients is not associated with an increased risk of cardiac tamponade, length of hospital stay or mortality.

**Relevance for patients:**

This study demonstrated that the use of DAPT in patients undergoing CABG as an urgent or emergency procedure following myocardial infarction is not associated with an increased risk of bleeding and has many associated benefits.

## 1. Introduction

Cardiac tamponade is a recognized early or delayed complication in open cardiac surgery, such as in 0.2% of coronary artery bypass grafting (CABG) and 8.4% of heart transplants, compromising recovery with associated morbidity and mortality [1-3]. Patients undergoing procedures for management or treatment of myocardial infarction will receive antiplatelet therapy to prevent further cardiovascular events. Aspirin therapy after CABG improves vein graft patency, particularly during the 1^st^ post-operative year, which is the most important determinant of long-term prognosis, and reduces major adverse cerebral and cardiovascular events [4,5]. Studies show significantly higher patency rates of vein graft 3 months after CABG in patients receiving dual antiplatelet therapy compared to those receiving aspirin monotherapy [5]. Antiplatelet and antithrombin therapy has been reported to reduce the risk of subsequent myocardial infarction in patients presenting with acute coronary syndrome (ACS) [6]. Benefits and risks of dual antiplatelet therapy (DAPT) in patients with ACS with medical therapy with/without coronary stent implantation (CSI) have been extensively discussed in the literature. However, only recently, more attention has been given to DAPT in ACS treated with CABG [5-7].

The main rationale for treating CABG patients with DAPT following myocardial infarction is for (a) pacification of the culprit unstable plaque, (b) prevention of subsequent spontaneous myocardial infarction, (c) increased saphenous vein patency, and (d) prevention of stent thrombosis in CSI patients before CABG [6]. First, the Clopidogrel in Unstable angina to prevent Recurrent ischemic Events (CURE) Trial found that the primary outcome had occurred in 14.5% of DAPT versus 16.2% of aspirin monotherapy patients before CABG (RR: 0.89, 95% CI: 0.71-1.11) [7]. Furthermore, the Platelet Inhibition and Patient Outcomes (PLATO) trial, comparing aspirin with either ticagrelor or clopidogrel, found a significant reduction in total mortality (9.7% vs. 4.7%), cardiovascular death (7.9% vs. 4.1%), and non-cardiovascular death (2.0% vs. 0.7%) [8].

Previous guidelines of the American College of Cardiology (ACC) and American Heart Association (AHA) did not include recommendations on DAPT in CABG patients. However, the 2016 update recommended the use of DAPT for at least 12 months in the management of patients undergoing urgent or emergency CABG [9,10]. There are potential risks associated with this therapy including bleeding which was identified as a significant risk. This may result in cardiac tamponade, necessitating re-opening of the thoracic cavity through emergency sternotomy with all the associated potential complications.

The purpose of this study was to determine the risk of bleeding following DAPT versus aspirin monotherapy in urgent and emergency CABG patients, influencing the potentially fatal sequelae of cardiac tamponade and overall mortality. The primary outcome was the reopening of the sternotomy wound for cardiac tamponade.

## 2. Methods

**Figure 1:**
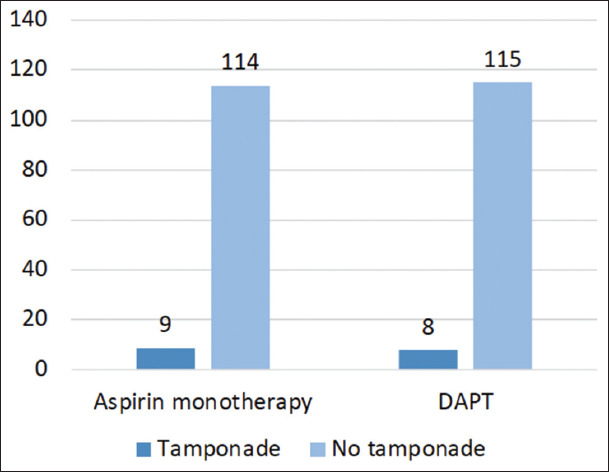
Tamponade as a complication in urgent/emergency CABG in AMT versus DAPT (P=0.802)

This study was reviewed and approved by the institutional Clinical Audit and Governance Committee of the Hull University Teaching Hospitals NHS Trust (IRB protocol number: 2018.012; approval date: 16/11/2018). Data were collected prospectively from patients admitted to Castle Hill Hospital, Hull, UK, a tertiary cardiac surgery center. Inclusion criteria were patients undergoing isolated urgent or emergency CABG procedures. Urgent is defined as patients undergoing surgery during the index admission following an ACS while emergency surgery is performed before the next elective theatre list. Patients undergoing multiple or other than CABG procedures were excluded from the study to minimize the confounding factors of complex operations. A population of 246 patients was identified among all urgent and emergency CABG procedures performed at the aforementioned center before and subsequent to the introduction of the 2016 guidelines. Patients in the aspirin monotherapy arm (*n*=123) were identified retrospectively between January 2015 and December 2015. Patients in the DAPT arm (*n*=123) were recruited prospectively from December 2016 to January 2018 (Figure 1). This chosen timeframe was utilized to recruit the maximum possible number of patients for inclusion in the study from the available database. The control monotherapy arm was chosen as recommended in the previous ACC/AHA guidelines [9]. Patients’ notes were obtained for retrospective data collection. The primary outcome was re-operation due to postoperative bleeding or tamponade. Secondary outcomes consisted of hospital stay, 30-day mortality and 1-year mortality. Both groups received the standard concentration of medicine. Data analysis was undertaken through IBM SPSS Statistics, v.26 (SPSS Inc., Chicago, IL, US). The collected data were a mixture of continuous and categorical variables. If continuous (age, length of hospital stays), evaluation of normality, equality of variance, outliers and missing data were performed. Continuous data are expressed as mean and standard deviation (SD) or median with interquartile range (Q1-Q3), if normally or non-normally distributed, respectively. Descriptive analysis was undertaken for nominal and ordinal data (number of coronary arteries affected, rates of cardiac tamponade, and incidence of mortality), which is expressed as numbers and percentages. An independent-sample t-test was used for continuous variables, when homogeneity was assumed with equal variances under Levene’s test. Where Levene’s test was violated, variance was not assumed. Mann–Whitney U-test was performed for non-normally distributed data. Pearson Chi-square test was undertaken to detect any statistical significance amongst categorical data. Two-tailed statistical significance was identified for *P*<0.05.

## 3. Results

All 246 patients identified as meeting the inclusion criteria were included in the study, with 123 patients in the DAPT arm and 123 in the control aspirin monotherapy arm (Figure 1).

Demographic and clinical details of the patients included in both arms of this study are summarized in Table 1 and the values in parentheses where presented, represent the range of the data. The numbers of male (AMT=101, DAPT=99) and female (AMT=22, DAPT=24) participants were comparable in both arms, and the median age in DAPT and AMT group was 71 (46-84) and 68 (37-85) years, respectively. Nevertheless, similar numbers of patients underwent single or multiple CABG procedures with no significant differences recorded (*P*=0.577) (Table 1).

**Table 1:** Patient demographics

	AMT (*n*=123)	DAPT (*n*=123)	*P* value
Age (years)	68 (37-85)	71 (46-84)	0.008*
Male (*n*=200)	101 (82.1)	99 (80.5)	-
Age (years)	68 (36-84)	70 (46-83)	0.029*
Age≥75 years	19 (15.5)	26 (21.1)	-
Weight (kg)	86.4 (±15.1)	84.2 (±13.4)	0.267*
Height (cm)	174.2 (±7.0)	172.9 (±7.0)	0.172*
BMI (kg/m^2^)	28.4 (±4.5)	28.2 (±4.0)	0.636*
Female (*n*=46)	22	24	-
Age (years)	69 (45-85)	73 (53-84)	0.130*
Age≥75 years	5 (4.1)	10 (8.1)	-
Weight (kg)	72.7 (±15.4)	69.5 (±11.3)	0.424*
Height (cm)	159 (±7.1)	159 (±5.8)	0.745*
BMI (kg/m^2^)	28.7 (±5.3)	27.8 (±4.5)	0.508*
Cardiovascular risk factors			
Smoker	83 (67.5)	79 (64.2)	-
Hypertension	104 (84.6)	89 (72.4)	-
Hypercholesterolemia	104 (84.6)	114 (92.7)	-
Diabetes mellitus	38 (30.9)	41 (33.3)	-
History			
Angina pectoris	93 (75.6)	105 (85.4)	-
Myocardial infarction	92 (74.8)	100 (81.3)	-
Congestive heart failure	12 (10)	16 (13)	-
Percutaneous coronary intervention	23 (18.7)	27 (22.0)	-
Transient ischemic attack or cerebrovascular event	17 (13.8)	10 (8.13)	-
Peripheral arterial disease	20 (16.3)	18 (14.6)	-
Number of distal coronary arteries affected			
1	4 (3.3)	5 (4.1)	0.577^†^
2	35 (28.5)	43 (35.0)	
3	65 (52.8)	60 (48.8)	
4	19 (15.4)	14 (11.4)	
5	0 (0.0)	1 (0.8)	

* Independent-samples Mann–Whitney U-Test.

† Pearson Chi-square test Unless otherwise indicated, data are rates, data are mean (±SD), median (Q1-Q3), or frequency (percentage)

Cardiac tamponade was observed in 9 (7.3%) and 8 (6.5%) of AMT and DAPT patients, respectively, with no significant statistical or clinical difference (*P*=0.802) (Table 2). All reoperations took place either in the Intensive Care Unit or in the theater and the median wait to reoperation was 533 (471.5**-**942.5) min in the AMT group and 656 (487-827) min in the DAPT group. It was noted that patients who were re-operated upon due to cardiac tamponade experienced a significant difference in length of hospitalization between the two arms of the study, AMT=10.1 (±2.9) versus DAPT=17.8 (±10.2) days (*P*=0.047). One patient from each group died as ICU in-patients within 30 days from the initial CAGB procedure due to multiorgan failure and hypoxic brain injury. In contrast, two more patients died in the subsequent year: One monotherapy patient on day 59 and one DAPT on day 58 following CABG surgery due to severe left ventricular dysfunction.

**Table 2:** Outcome evaluation

	AMT	DAPT	*P* value
Number of reoperations for cardiac bleeding or tamponade			
Tamponade	9 (7.3%)	8 (6.5%)	0.802^†^
No tamponade	114 (92.7%)	115 (93.5%)	
Time in minutes to reoperation in patients with tamponade (range)	533 (471-942)	656 (487-827)	0.713^#^
Hospital stay (days)			
Tamponade	10.1 (±2.9)	17.8 (±10.2)	0.047^#^
No tamponade	14 (15-59)	12 (5-58)	0.144*
Hospital stay in ICU (hours)			
Tamponade	49.8 (26.5-78.6)	25.3 (22.2-69.3)	0.273^#^
No tamponade	26 (22-47.5)	114.8 (78.2-136.9)	0.831^#^
Mortality			
30-day	1 (0.8)	1 (0.8)	0.078^†^
1-year	1 (0.8)	1 (0.8)	
Alive	121 (98.4)	121 (98.4)	

# Independent t-test.

* Independent-samples Mann–Whitney U-Test.

† Pearson Chi-Square test. Unless otherwise indicated, data are mean (±SD), median (Q1-Q3), or frequency (percentage)

In addition, overall, there were no significant differences in blood products requirement between the treatment arms (Table 3). Nevertheless, tamponade DAPT patients required significantly higher total packed cells compared to non-tamponade (*P*=0.004), and tamponade patients in both groups were administered significantly higher total blood expanders and experienced significantly higher loss of blood volume compared to the corresponding non-tamponade subjects (*P*<0.001). Of note, although not statistically significant, tamponade DAPT patients were administered a higher volume of total platelets than monotherapy comparisons, in association with significant outliers.

**Table 3:** Blood products

	AMT		DAPT		*P* value
	
	*n*	Value (range)	*n*	Value (range)	
Hemoglobin before procedure				
Tamponade	10	136.5 (133.3-139.3)	7	135.1 (121.8-147.3)	0.051^#^
No tamponade	112	139.5 (126.3-149.7)	114	113.2 (96.4-134.1)	0.390^#^
Lowest hemoglobin				
Tamponade	10	35.5 (34.2-36.4)	7	35.1 (33.6-36.3)	0.710^#^
No tamponade	112	35.8 (34-36.5)	114	35.6 (34.3-36.2)	0.563^#^
Total autologous blood (mL)				
Tamponade	9	458 (374-568)	7	608 (426-765)	0.221^#^
No tamponade	86	478 (309-636)	112	474 (306-788)	0.035^#^
Total packed cells (mL)				
Tamponade	9	600 (577-983)	7	1610 (849-2144)	0.130^#^
No tamponade	25	576 (304-1144)	35	290 (259-618)	0.586^#^
Total fresh frozen plasma (mL)				
Tamponade	4	1117.(702-1123)	4	1071(566-1086)	0.320^#^
No tamponade	4	828 (538-1120)	11	1074 (1005-2647)	0.506^#^
Total cryoprecipitate (mL)				
Tamponade	0	-	2	573	-
No tamponade	2	363	1	430	-
Total platelets (mL)				
Tamponade	5	487 (322-527)	7	511 (299-1635)	0.438^#^
No tamponade	10	406 (289-567)	110	319 (284-506)	0.432^#^
Total blood expanders (mL)				
Tamponade	9	2250 (1670-2890)	7	2407 (1107-3210)	0.734^#^
No tamponade	101	1000 (575-1250)	112	1000 (500-1500)	0.729^#^
Total blood loss (mL)				
Tamponade	9	1685(1280-1980)	10	2105 (1985-2524)	0.157^#^
No tamponade	111	410 (325-695)	113	482 (333-665)	0.188^#^

# Independent Student’s *t*-test. Unless otherwise indicated, data are mean (±SD), median (Q1-Q3), or frequency (percentage) and *P*-values refers to comparison between outcome parameter values

## 4. Discussion

This is the first study to demonstrate the safety of DAPT versus aspirin monotherapy in urgent and emergency CABG patients, utilizing cardiac bleeding or tamponade as a primary outcome.

In accordance with previous studies, all bleeding events occurred within 24 h from the initial CABG procedure, with only one fatal bleed occurring in the aspirin group during the re-operation for tamponade [8]. No delay in re-operating on DAPT patients was observed compared to the aspirin monotherapy group. However, a non-statistically significant clinical difference was observed in intraoperative blood loss volume between aspirin and DAPT, which was higher in the latter, and a statistically higher volume of lost blood was described in DAPT tamponade patients compared to non-tamponade (*P*<0.001). This was associated with a similar pattern observed as per administered volume of blood expanders in the same groups (*P*<0.001). It is speculated that this may have resulted from a delay in the tamponade operation to allow the antiplatelet effect to wear off, thereby increasing the proneness to intraoperative bleeding. As ascertained from the literature, it is generally-accepted that supplementary antithrombotic therapy results in increased bleeding risk [5]. Nevertheless, intraoperative platelet administration was higher in re-operated DAPT patients.

Intraoperative treatment with antifibrinolytic agents, such as aprotinin and tranexamic acid, was reported to reduce perioperative bleeding in elective CABG procedures but also reported to be associated with increased morbidity and mortality [11]. Nevertheless, this study cannot state that the increased intraoperative bleeding or use of blood products is associated with the DAPT therapy or lack of administration of aprotinin. Aprotinin was not available in our department until 2019 and all patients received tranexamic acid infusion. Thromboelastography (TEG) was used during the study period to guide transfusion of blood products in patients undergoing CABG. It has a role in the routine management of CABG patients to improve clinical outcomes and to reduce potential risks associated with transfusions and total CABG cost [12,13]. Furthermore, TEG-based coagulation management decreases significantly the rates of re-exploration, post-operative thromboembolic events, and acute kidney injury [14].

### 3.1. Limitations of the study

Despite the observed increased intraoperative bleeding in DAPT patients, no significant differences were observed in total hospital stay, total hours spent in ICU and mortality between aspirin monotherapy and DAPT. Nevertheless, accounting for a relatively small cohort size, this study does not exclude the possibility of type II error. Therefore, it is recommended that further research on larger sample sizes at different centers be conducted to address this potential limitation. It is also noted that the two groups were operated on during different although subsequent time frames which may raise the question of changing perioperative care, no other changes were introduced into our practice apart from the DAPT. To analyze the effect of other risk factors, we need to perform the propensity matching to identify two similar groups which is difficult as total number of patients are not enough and also, its beyond the scope of this paper.

In conclusion, from this study, it is demonstrated that the use of DAPT in this cohort of patients undergoing CABG as an urgent or emergency procedure following myocardial infarction is not associated with an increased risk of bleeding and has many associated benefits.
